# The Age at Diagnosis of Autism Spectrum Disorder in Children in Japan

**DOI:** 10.1155/2018/5374725

**Published:** 2018-05-07

**Authors:** Shigeki Kurasawa, Kiyomi Tateyama, Ryoichiro Iwanaga, Taro Ohtoshi, Ken Nakatani, Katsushi Yokoi

**Affiliations:** ^1^Department of Rehabilitation Sciences, Kansai University of Welfare Sciences, 3-11-1 Asahigaoka, Kashiwara 582-0026, Japan; ^2^Department of Occupational Therapy, School of Comprehensive Rehabilitation, College of Health and Human Sciences, Osaka Prefecture University, 3-7-30 Habikino, Habikino, Osaka 583-8555, Japan; ^3^Department of Health Sciences, Nagasaki University Graduate School of Biomedical Sciences, 1-12-4 Sakamoto, Nagasaki 852-8523, Japan; ^4^Department of Occupational Therapy, Faculty of Health Sciences, Morinomiya University of Medical Sciences, 1-26-16, Nankoukita, Suminoe-ku, Osaka 559-0024, Japan

## Abstract

**Background:**

No large-scale study of the timing of autism spectrum disorder (ASD) diagnosis has been performed in Japan to date. The aim of this study was to examine sex differences and annual trends in age at diagnosis of ASD using clinical data.

**Methods:**

Clinical data for children aged less than 18 years diagnosed with ASD between January 1, 2009, and December 31, 2013, and in whom follow-up was possible 1 year after diagnosis, were extracted.

**Results:**

The mean age at ASD diagnosis was 7.2 ± 4.2 years and the mode age was 3 years. No sex difference was observed for age at diagnosis (*p* = 0.157). An annual trend of earlier diagnosis was observed when fiscal years were compared (*p* < 0.001).

**Conclusion:**

This study highlighted the need to develop and provide appropriate early intervention methods and services for ASD children in Japan.

## 1. Introduction

In the International Statistical Classification of Diseases and Related Health Problems (ICD-10), pervasive developmental disorder (PDD) is classified into childhood autism, atypical autism, Rett syndrome (RS), overactive disorder associated with mental retardation and stereotyped movements, Asperger's syndrome, other childhood disintegrative disorders, and PDD-unspecified. Meanwhile, the Diagnostic and Statistical Manual of Mental Disorders Fifth Edition (DSM-5) considers PDDs, with the exception of RS, to be synonymous with autism spectrum disorder (ASD). The prevalence of ASD is approximately 1% and an upward trend has been reported [1–4]. Many reports are skeptical that the increase in ASD is the result of an increase in risk factors, and many are of the opinion that the increase is the result of changes to diagnostic criteria and a younger age at diagnosis [1, 3]. Many studies of the increase in ASD are underway, and research into therapeutic interventions is also ongoing. Nonpharmacological therapies for ASD include approaches based on applied behavior analysis (e.g., the Lovaas approach), structured teaching (e.g., Treatment and Education of Autistic and related Communication Handicapped Children), and targeted skill-based intervention (e.g., Picture Exchange Communication System). All of these therapies have confirmed evidence levels [1, 5], with some interventions reported to be more effective when implemented early [1, 6, 7], leading to increased interest in the early diagnosis of ASD.

There have been several reviews and large-scale study reports of age at ASD diagnosis [8–10]. However, studies of age at ASD diagnosis have major problems. The first is the potential for the residential area of the child to have an effect on the timing of ASD diagnosis [8, 11]. It is easily conceivable that the timing of ASD diagnosis is affected by the medical or healthcare system of the area. Studies of age at ASD diagnosis therefore need to examine age by country and region. The second problem is the uncertainty regarding the existence of a sex difference in the timing of ASD diagnosis. Some reports have suggested that the timing of ASD or AS diagnosis is delayed in girls without mental and behavioral disorders [2, 9]. However, no large-scale survey of the timing of diagnosis has been conducted in Japan. The third problem is whether annual trends can be seen in the age at ASD diagnosis. World Autism Awareness Day was established at the United Nations General Assembly in 2007 and has likely increased social awareness of ASD. Tools have also been developed to allow for the early diagnosis of ASD [4, 12]. These changes could cause annual trends in the age at ASD diagnosis.

The aim of this study was to examine sex differences and annual trends in age at diagnosis of ASD using clinical data.

## 2. Materials and Methods

### 2.1. Materials and Methods

#### 2.1.1. Subject Data and Extraction of Samples

**Figure 1:**
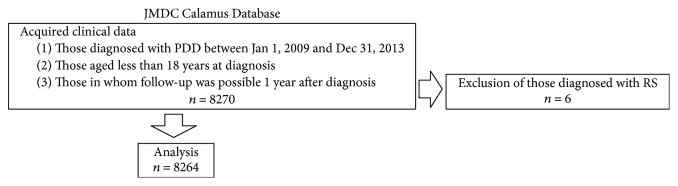
Flowchart of the process from data sample extraction to analysis.

Clinical data were acquired from Japan Medical Data Center Co., Ltd. (JMDC), which is Japan's largest provider of clinical data. The data are a consolidation of all clinical data of insured persons held by multiple contracted health insurance societies, thereby facilitating the understanding of information such as consultations and transfers by multiple medical institutions without omissions. The acquired clinical data included the basic attributes of patients, the type of medical institution, the disease name (ICD-10 classification codes), and the content of therapy.  Table 1 shows the characteristics of the JMDC data including the coverage. JMDC clinical data included data from 80% or more of hospitals and clinics in Japan from 2009 through 2013. A flowchart of the process from sample extraction to analysis is presented in Figure 1. In the present study, the data that met all of the following conditions were extracted from among the clinical data held by the JMDC: (1) those diagnosed with PDD between January 1, 2009, and December 31, 2013; (2) those aged less than 18 years at diagnosis; and (3) those in whom follow-up was possible 1 year after diagnosis. At the end, those with RS (*n* = 6) were excluded and 8264 samples were used in the final analysis.

### 2.2. Analysis Methods

Descriptive statistics of the acquired samples were used to understand the basic attributes of the subjects, the diagnosing medical institution, and the clinical department, after which sex differences in age at diagnosis were examined by type of ASD using the Mann-Whitney* U* test. Thereafter, annual trends in age at ASD diagnosis were examined by the Kruskal-Wallis test. The level of significance was set at 5%, and IBM SPSS ver. 24.0 was used for statistical analyses.

### 2.3. Research Ethics

The clinical data used in the present study were provided by JMDC with the consent of the JMDC ethical review board. This study was also approved by the Research Ethics Committee of Kansai University of Welfare Sciences (approval number: 15-01).

## 3. Results

The basic attributes of the subjects are presented in Table 2. The mean age at ASD diagnosis was 7.2 ± 4.2 years and the mode age was 3 years. The male-to-female ratio was 76.1% males and 23.9% females. The diagnostic criteria for ASD using ICD-10 were as follows: childhood autism (33.6%), atypical autism (2.9%), Asperger's syndrome (9.4%), other pervasive developmental disorder (0.7%), and PDD-unspecified (47.6%). Of the 5.9% of subjects who were diagnosed as having overlapping conditions with PDD during their first consultation, 72% were diagnosed with childhood autism and PDD-unspecified (data not shown). Furthermore, other forms of childhood disintegrative disorder and overactive disorder associated with mental retardation and stereotyped movements were not included in the dataset. The diagnosing medical institution was a medical office in most cases (52.9%), followed by a national hospital or public hospital (24.1%), university hospital (4.7%), and other medical institutions (18.3%). The clinical department was pediatrics for 36.2% of the subjects, neuropsychiatry for 28.7%, and internal medicine for 24.5%. These three clinical departments made approximately 90% of diagnoses.

Table 3 shows the sex differences in age at ASD diagnosis. The mean age at diagnosis was 7.18 years in males and 7.42 years in females, and there was no significant difference (*p* = 0.157). The median age was 6.0 years for both males and females, and the modal age was 3 years.

**Figure 2:**
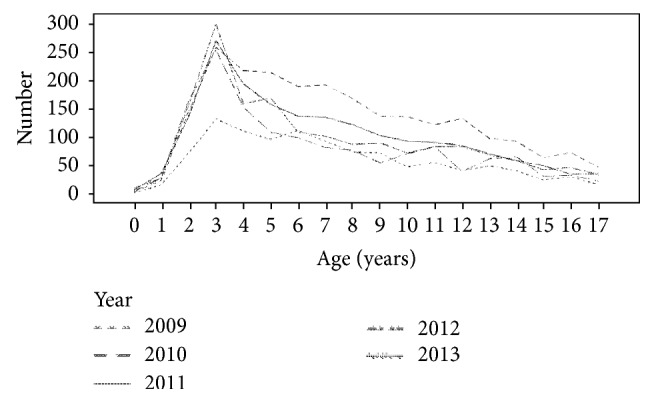
Annual trends in age at diagnosis and patient numbers.

Figure 2 shows the sample size at the age at diagnosis for each fiscal year. The mode age was 3 years in all fiscal years, and a tapering in the frequency of occurrence was observed as the age increased beyond 3.

**Figure 3:**
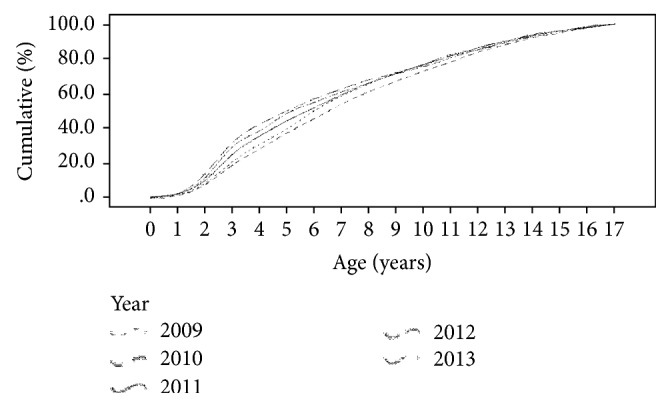
Annual trends in age at diagnosis and cumulative percentage of patients.

Table 4 shows the results of a comparison between the age at diagnosis and fiscal year. The age at diagnosis for each fiscal year was as follows: 7.8 years in 2009, 8.2 years in 2010, 7.5 years in 2011, 7.1 years in 2012, and 6.3 years in 2013. The median age at diagnosis was 7.0 years in 2009, 8.0 years in 2010, 7.0 years in 2011, 5.0 years in 2012, and 4.0 years in 2013. A statistical difference was observed when the age at diagnosis was compared with fiscal year using the Kruskal-Wallis test (*p* < 0.001). Comparison of fiscal years in pairs revealed the following: the age at diagnosis in 2009 was significantly higher than 2012 and 2013; the age at diagnosis in 2010 was significantly higher than 2011, 2012, and 2013; and the age at diagnosis in 2012 was significantly higher than 2013. A trend towards younger age at diagnosis in recent years was also observed in Figure 3, which shows the frequency of occurrence as cumulative percentages.

## 4. Discussion

The basic attributes of the subjects showed a male-to-female ratio of 3 : 1. The male-to-female ratio of ASD is generally considered to be 4 : 1 [2]. However, this finding needs to be examined carefully to establish if it is specific to Japan. The criteria for sample extraction in the present study differed from normal methods of calculating prevalence. Samples were extracted longitudinally in the present study, which means that potential bias resulting from a period effect cannot be ruled out. However, a study in Yokohama City, Japan, that used ICD-10 diagnostic criteria calculated a male-to-female ratio of 2.5 : 1 [13], which was close to that of the present study. The present study was a large-scale study that accumulated data over a 5-year period; therefore, the male-to-female ratio of ASD observed in the present study can be generalized. No sex difference was observed in the age at ASD diagnosis, and the mode age of both males and females was 3 years. The mode age for each fiscal year was also 3 years. Under Japanese maternal and child health practice, health diagnoses are performed at 18 months and 3 years of age, when the majority of infants undergo screening by a physician. Our data showed that the peak age at diagnosis was the age of 3, highlighting the contribution of ASD screenings during infant health checkups in Japan. However, the number of diagnosed patients after the peak age of 3 showed a gradual declining curve, which cannot be underestimated. Concern about undiagnosed cases of Asperger's syndrome and high-functioning autism has been reported [14]. Consequently, there is a need to examine the association between delays in ASD diagnosis and the ASD subtypes. A decreasing trend in the age at diagnosis was observed when the age at diagnosis was compared for each fiscal year. This may be due to increased social awareness of ASD, but it might also be related to the development of effective diagnostic ASD screening tests and costs [4, 12]. On the other hand, early diagnosis suggests the need to develop and provide more effective early-stage ASD rehabilitation interventions. Infants with ASD have comorbid sleep disorders associated with disruptions in the autonomic nervous system and hypersensitivity [15, 16]. Sleep disorders in ASD may affect the school life of the children in areas such as social interaction, academic achievement and behavioral problems [15, 17]. However, there is no sufficient evidence yet of effective interventions addressing sleeping disorders of infants with ASD. Furthermore, there is not much clear evidence of the therapeutic effects of the various traditional nonpharmacological therapies on infants [1]. The findings of the present study highlighted the need to develop and provide appropriate, early intervention methods, and services for children with ASD.

## 5. Limitations

This study has several limitations. The first was the inability to conduct a simple comparison of data with other previous studies since the present study, unlike cohort studies, analyzed the data of patients who voluntarily sought consultations. There might have also been a bias influencing parents' decision-making during consultations of the children in this study. The second limitation was that the sample used in this study included no regional data such as place of residence. It was therefore not possible to examine factors including differences in regional medical services, and the possibility of bias in the findings related to age at diagnosis cannot be ruled out. The third limitation was that it was not possible to gain an accurate understanding of the total population within the follow-up period. It was therefore not possible to consider the prevalence of ASD in the process of examining the incidence by fiscal year. However, the total specific birth rate in Japan from 1990 to 2013 was within the range of 1.26–1.57, indicating no major variation [18]. The findings of this study are therefore considered reasonable.

## 6. Conclusions

The age at ASD diagnosis was examined by the subtype of ASD using Japanese clinical data. There was no sex difference in age at ASD diagnosis. An annual trend of earlier diagnosis was observed when fiscal years were compared. The findings of the present study highlighted the need to develop and provide appropriate, early intervention methods, and services for children with ASD.

## Figures and Tables

**Table 1:** Characteristics of JMDC clinical data.

	Number of medical institutions^†1^		No. of JMDC-contracted medical institutions		JMDC coverage	
	Hospital^†2^	Clinic^†3^	Hospital^†2^	Clinic^†3^	Hospital^†2^	Clinic^†3^
2009	8,739	99,635	7,398	81,779	84.7%	82.1%
2010	8,670	99,824	7,556	84,558	87.2%	84.7%
2011	8,605	99,547	7,641	87,127	88.8%	87.5%
2012	8,565	100,152	7,658	86,582	89.4%	86.5%
2013	8,540	100,528	7,529	84,384	88.2%	83.9%

^†1^Number of medical facilities according to a Ministry of Health, Labour and Welfare survey. ^†2^Medical facilities with 20 or more beds. ^†3^Medical facilities with 0–19 beds.

**Table 2:** Basic attributes of the subjects (*n* = 8264).

Age at diagnosis in years	
Modal age	3.0
Median (25th–75th percentile)	6.0 (4.0–10.0)
Mean ± SD	7.2 ± 4.2
Sex *n* (%)	
Male	6285 (76.1)
Female	1979 (23.9)
Autism spectrum disorder^†1^ *n* (%)	
Childhood autism	2778 (33.6)
Atypical autism	241 (2.9)
Asperger's syndrome	773 (9.4)
Other pervasive developmental disorder	56 (0.7)
Pervasive developmental disorder, unspecified	3931 (47.6)
Dual and multiple diagnosis	485 (5.9)
Medical facilities *n* (%)	
National hospital, public hospital	1989 (24.1)
University hospital	392 (4.7)
Other hospital	1509 (18.3)
Medical office	4374 (52.9)
Diagnosis and treatment department *n* (%)	
Paediatrics	2991 (36.2)
Neuropsychiatry	2368 (28.7)
Internal medicine	2025 (24.5)
Orthopedics	167 (2.0)
Obstetrics and gynecology	114 (1.4)
Otolaryngology	46 (0.6)
Radiology	44 (0.5)
Ophthalmology	38 (0.5)
Neurosurgery	22 (0.3)
Urology	17 (0.2)
Others	432 (5.2)

^†1^Subtype of PDD using the first date of diagnosis (exclusion of RS).

**Table 3:** Sex differences in age (years) at ASD diagnosis.

	*n*	Modal age	Median (25th–75th)	Mean ± SD	*p* value
Male	6285	3.0	6.0 (4.0–10.0)	7.2 ± 4.2	0.157
Female	1979	3.0	6.0 (3.0–11.0)	7.4 ± 4.4	

*Note*. Mann-Whitney *U* test.

**Table 4:** Annual trends in age (years) at ASD diagnosis.

Year	Mean ± SD	Median	*p* for difference				
		(25–75%ile)	2009	2010	2011	2012	2013
2009	7.8 ± 4.3	7.0 (4.0–11.0)	—	NS	NS	*∗*	*∗∗*
2010	8.2 ± 4.3	8.0 (4.0–12.0)	—	—	*∗∗*	*∗∗*	*∗∗*
2011	7.5 ± 4.5	7.0 (3.0–11.0)	—	—	—	NS	*∗*
2012	7.1 ± 4.6	5.0 (3.0–11.0)	—	—	—	—	NS
2013	6.3 ± 4.3	4.0 (3.0–9.0)	—	—	—	—	—

*Note.* Kruskal-Wallis test (*p* < 0.001); Pairwise Kruskal-Wallis test; NS: not significant; ^*∗*^*p* < 0.05; ^*∗∗*^*p* < 0.01.
